# Timing of Maternal Exposure to a High Fat Diet and Development of Obesity and Hyperinsulinemia in Male Rat Offspring: Same Metabolic Phenotype, Different Developmental Pathways?

**DOI:** 10.1155/2013/517384

**Published:** 2013-05-13

**Authors:** Graham J. Howie, Deborah M. Sloboda, Clare M. Reynolds, Mark H. Vickers

**Affiliations:** ^1^Liggins Institute and Gravida: National Centre for Growth and Development, University of Auckland, 2-6 Park Avenue, Grafton, Auckland 1010, New Zealand; ^2^Department of Biochemistry and Biomedical Sciences, McMaster University, Hamilton, ON, Canada L8S 4L8

## Abstract

*Objective.* Offspring born to mothers either fed an obesogenic diet throughout their life or restricted to pregnancy and lactation demonstrate obesity, hyperinsulinemia, and hyperleptinemia, irrespective of their postweaning diet. We examined whether timing of a maternal obesogenic diet results in differential regulation of pancreatic adipoinsular and inflammatory signaling pathways in offspring. *Methods.* Female Wistar rats were randomized into 3 groups: (1) control (CONT): fed a control diet preconceptionally and during pregnancy and lactation; (2) maternal high fat (MHF): fed an HF diet throughout their life and during pregnancy and lactation; (3) pregnancy and lactation HF (PLHF): fed a control diet throughout life until mating, then HF diet during pregnancy and lactation. Male offspring were fed the control diet postweaning. Plasma and pancreatic tissue were collected, and mRNA concentrations of key factors regulating adipoinsular axis signaling were determined. *Results.* MHF and PLHF offspring exhibited increased adiposity and were hyperinsulinemic and hyperleptinemic compared to CONT. Despite a similar anthropometric phenotype, MHF and PLHF offspring exhibited distinctly different expression for key pancreatic genes, dependent upon maternal preconceptional nutritional background. *Conclusions.* These data suggest that despite using differential signaling pathways, obesity in offspring may be an adaptive outcome of early life exposure to HF during critical developmental windows.

## 1. Introduction

Early life events contribute substantially to the likelihood of an individual becoming obese, although underlying mechanisms are not well understood. Obesity in women of reproductive age (15 to 44 years) is increasing rapidly, and up to 50% of women in this age range in the USA are now either overweight or obese [1]. This has translated to an exponential increase in the prevalence of obesity during pregnancy with up to 20% of women entering pregnancy with a BMI which would define them as obese [2]. Obesity in pregnancy increases the risks for complications of pregnancy including miscarriage, hypertension, and gestational diabetes [3–5]. Furthermore, it is now well established that maternal obesity leads to an increased risk of obesity and metabolic and cardiovascular disorders in offspring [6–9]. In view of the rising prevalence of obesity in pregnancy and its association with adverse maternal and offspring outcomes, there is a great deal of interest in understanding the mechanistic pathways that link maternal obesity and excess maternal nutrition to increased risk of disease in childhood and beyond [10–13].

Clinical and experimental studies have consistently shown that maternal obesity predicts obesity, associated metabolic comorbidities, and reproductive disorders in adult offspring [2, 14–16], often underpinned by a proinflammatory status. However, there is a need to understand the relative contributions and interactions between maternal prepregnancy obesity and a gestational obesogenic environment on subsequent health and well-being of offspring. We have previously shown that obesity in adult rat offspring arising as a consequence of maternal high fat (HF) nutrition was independent of the pre-conceptional maternal diet [14]. We have shown that offspring of mothers fed an HF diet restricted to pregnancy and lactation compared to those born to mothers fed a HF diet prior to conception and throughout pregnancy and lactation both exhibit a remarkably similar obesogenic phenotype, characterised by increased fat mass, hyperinsulinemia, and hyperleptinemia. These data suggested that despite a common obesity phenotype, differential mechanisms based on the timing of maternal exposure to HF nutrition may underpin pancreatic adipoinsular axis dysfunction in these offspring [17–19]. We therefore investigated key components of the leptin-insulin (adipoinsular) and inflammatory signaling pathways in pancreatic tissue of offspring born to mothers fed a high fat diet during pregnancy in the presence or absence of pre-conceptional HF diet-induced obesity.

## 2. Methods

The rat model of maternal high fat nutrition has been described by our group previously [14, 15, 20–24]. In brief, male and female Wistar rats were acquired at a weaning age (22 days, Vernon Jansen Unit, University of Auckland) and housed two per cage under standard conditions with a 12:12 light dark cycle and free access to water. Females were weight-matched and assigned to receive either standard rat chow (*n* = 16, 5% fat, Diet 2018, Harlan Teklad, Oxon) or a high fat (HF) diet (*n* = 8, 24% fat, D12451, Research Diets, NJ, USA) to be fed ad libitum for the duration of the trial. Males were fed standard chow ad libitum for the duration of the premating period. Body weights were recorded every 3 days until postnatal day 120.

At postnatal day 110, body composition in females was quantified using dual energy X-ray absorptiometry (DEXA, Lunar Prodigy, GE Medical Systems, Madison, WI, USA). At postnatal day 120, females were time-mated using an estrus cycle monitor (Model EC40, Fine Science Tools, CA, USA). Upon confirmation of mating, three maternal dietary groups were established: (1) controls (CONT): females fed a standard chow diet throughout their life and maintained on a standard chow diet throughout pregnancy and lactation; (2) maternal high fat diet (MHF): females fed an HF diet throughout their life and maintained on the HF diet throughout pregnancy and lactation; and (3) pregnancy + lactation HF diet (PLHF): females fed a standard chow diet until conception and then an HF diet throughout pregnancy and lactation. All pregnant dams were weighed and had food intakes measured daily throughout pregnancy. Following birth, pup weights and body lengths were recorded and litter size was randomly adjusted to 8 pups per litter to ensure standardized nutrition until weaning. Body weights and food intakes of dams were measured throughout the lactation period, and offspring were weighed every three days until weaning.

At weaning (d22), offspring were housed 2 per cage (2 per litter/maternal background) and fed a chow diet until the end of the trial. At postnatal day 150, body composition was measured in male offspring (*n* = 10–18 per group) by DEXA. At postnatal day 160, animals were fasted overnight and killed by decapitation following anaesthesia with sodium pentobarbitone (60 mg/kg, IP). Blood was collected into heparinized tubes, centrifuged and plasma supernatant stored for future analysis. Whole pancreata were removed and immediately snap-frozen in liquid nitrogen for later molecular analysis. All animal experiments were approved under guidelines of the Animal Ethics Committee at the University of Auckland (Approval R652).

### 2.1. Plasma Analysis

Fasting plasma leptin and insulin concentrations were analysed using commercial rat-specific ELISAs (CrystalChem 90040 and 90060, resp., Downers Grove, IL, USA). Fasting whole blood glucose concentrations were measured using a glucose meter at the time of tissue collection (d160) (Roche AccuChek). Plasma tumor necrosis factor (TNF-) alpha, interleukin (IL-) 1*β* and IL-6 were measured using commercially available ELISAs (Quantikine kits, RTA00, RLB00, and R6000B, resp., R&D Systems Europe, Abingdon, UK). The homeostatic model assessment (HOMA) method was used as a measure of insulin resistance (IR) and beta-cell function (HOMA-IR = plasma glucose × insulin/22.5) [25, 26].

### 2.2. Gene Expression

Quantitative PCR methods have been described previously in detail [23, 27]. Briefly, whole pancreas tissue was disrupted using a homogenizer, and total RNA was extracted using commercially available kits (Qiagen Mini-Prep; catalogue number 80204) according to the manufacturer's instructions. RNA integrity and quality were assessed using a NanoDrop spectrophotometer (ND-1000; BioLab Ltd.) using NanoDrop software (version 3.1.2) where sample ratio A260/280 and A260/230 were ~2.0, and intact bands were visualised by gel electrophoresis. Complimentary (c) DNA was synthesised from RNA (5 *μ*g) by reverse transcription PCR using MMLV-RT (Invitrogen, CA, USA). Quantitative PCR assays were performed in 15 *μ*L reaction volumes using EXPRESS SYBR GreenER (Invitrogen), and fluorescence was measured and quantified using an ABI-7900HT Ver.2.3 Sequence Detection System (Applied Bio Systems, CA, USA). The qPCR thermal profile for amplification of all genes was as follows: melting at 95°C for 15 secs, followed by annealing/extending at 60°C for 1 min, for 40 cycles. 

Primers were obtained either commercially or designed *de novo* (Table 1) using NCBI “Primer-BLAST”. A standard curve was generated using 6-fold serial 1 : 5 dilutions of pooled stock cDNA. All samples were assayed in triplicate and only those assays displaying a single PCR peak in the melt-curve analysis were used. All qPCR results were normalized against the geometric mean of housekeeper genes cyclophilin and HPRT, according to the geNorm method of Vandesompele et al. [28]. TNF, IL-1R1, and CD68 (and respective controls HPRT and cyclophilin) were analysed using Taqman gene expression assays. To control for between-sample variability, mRNA levels were normalized to the geometric mean of the controls. The ΔCt for each treatment sample was compared to the mean ΔCt for control samples using the relative quantification 2-(ΔΔCt) method to determine fold-change [29].

### 2.3. Statistics

All data were analysed by analysis of variance (ANOVA) with maternal diet as factor with post hoc testing to compare groups of interest as required (Holm-Sidak). Data with nonnormal distributions were log transformed if required. Analysis was performed using StatView statistical software (SAS, USA). Litter was used as a covariate with each litter considered a single biological replicate. Data are presented as means ± SEM unless otherwise stated.

## 3. Results

### 3.1. Maternal Weights and Body Fat Mass

A pre-conceptional HF diet (MHF) resulted in a significant increase in female body weight (chow 270 ± 5 g, MHF 310 ±  8, *P* < 0.05) and total fat mass compared to chow fed animals (chow 17.8 ± 0.7%, MHF 27.7 ± 1.7, *P* < 0.0001). At the start of pregnancy, there were no differences in body weights between females randomly assigned to CONT and PLHF groups (CONT 270 ± 8 g, PLHF 269 ± 7). Total caloric intake was similar between CONT and MHF groups but was significantly increased in PLHF dams during the first 10 days of pregnancy (average caloric intake: CONT 0.23 ± 0.004 kcal/gBW/day, MHF 0.23 ± 0.004, PLHF 0.32 ± 0.01, *P* < 0.05 for PLHF versus CONT and MHF); thereafter, caloric intake was similar to that of CONT and MHF dams. 

### 3.2. Body Weight and Body Composition in Male Offspring

As reported previously [14], MHF and PLHF pups had slightly but statistically significantly reduced body weights and were hypoinsulinemic and hypoleptinemic compared to CONT offspring at birth. MHF and PLHF neonates displayed rapid catch-up growth and were heavier than CONT neonates at the time of weaning (day 22: CONT 61.6 ± 0.7 g, MHF 70.5 ± 0.9, PLHF 64.2 ± 1.2).

**Figure 1:**
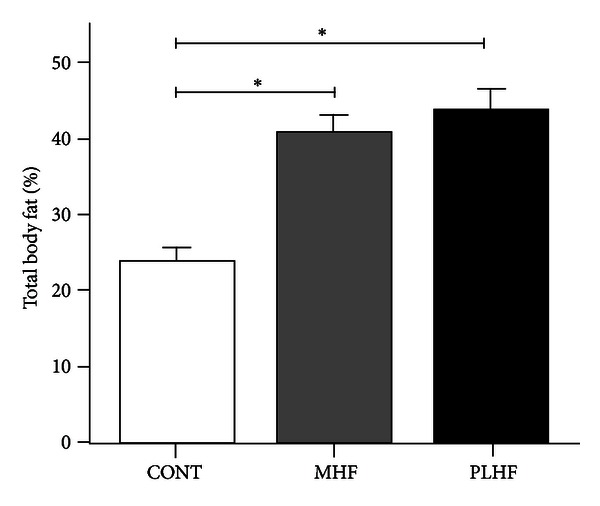
Total body fat mass (percent) as quantified by DEXA scan in adult CONT, MHF, and PLHF offspring. Data are means ± SEM, *n* = 10–12 per group. *P < 0.05.

Final adult body weights were increased in MHF and PLHF male offspring compared to CONT (CONT 562 ± 9 g, MHF 635 ± 18, PLHF 628 ± 17) [14]. MHF and PLHF adult male offspring had significantly increased total body fat mass at day 150 compared to CONT (Figure 1). There were no differences in total fat mass between MHF and PLHF adult offspring. 

### 3.3. Plasma Analyses

**Figure 2:**
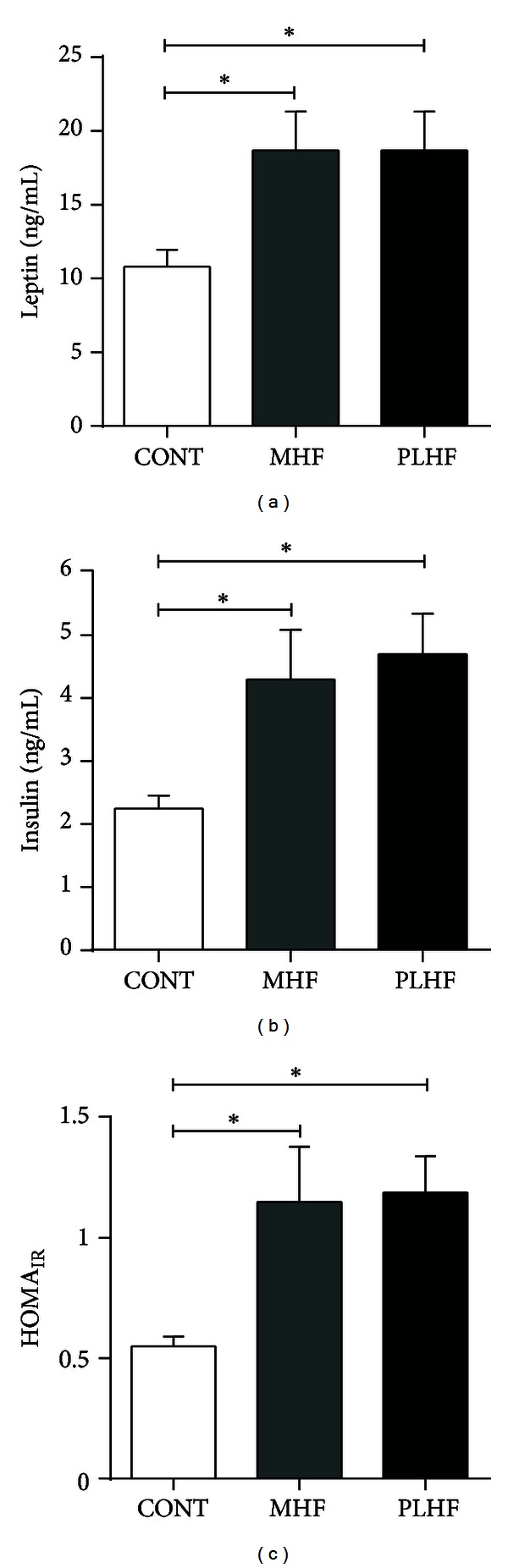
Fasting plasma leptin (a), insulin (b), and homeostatic model assessment (HOMA) to quantify insulin resistance and beta-cell function (c) in adult CONT, MHF, and PLHF offspring. Data are means ± SEM, *n* = 10–18 per group. *P < 0.05.

MHF and PLHF adult male offspring were hyperleptinemic and hyperinsulinemic compared to CONT (Figures 2(a) and 2(b)). Fasting blood glucose levels were similar between groups (CONT 5.6 ± 0.2 mmol/L, MHF 5.7 ± 0.3, PLHF 5.7 ± 0.2). Glucose: insulin ratios were significantly (*P* < 0.001) reduced in MHF and PLHF male offspring compared to control (CONT 2.5 ± 0.2, MHF 1.3 ± 0.1, PLHF 1.2 ± 0.1) and offspring exhibited increased insulin resistance using HOMA analysis (Figure 2(c)). Plasma TNF-*α* levels were not significantly different between the groups (CONT 3.6 ± 0.9 pg/mL, MHF 6.7 ± 1.9, PLHF 5.1 ± 1.9). Plasma IL-1*β* levels were significantly increased in MHF (*P* < 0.05) offspring compared to control (CONT 31.9 ± 0.7 pg/mL, MHF 38.3 ± 2.8, PLHF 36.1 ± 2.2) but were not different between CONT and PLHF offspring.

### 3.4. Gene Expression

**Figure 3:**
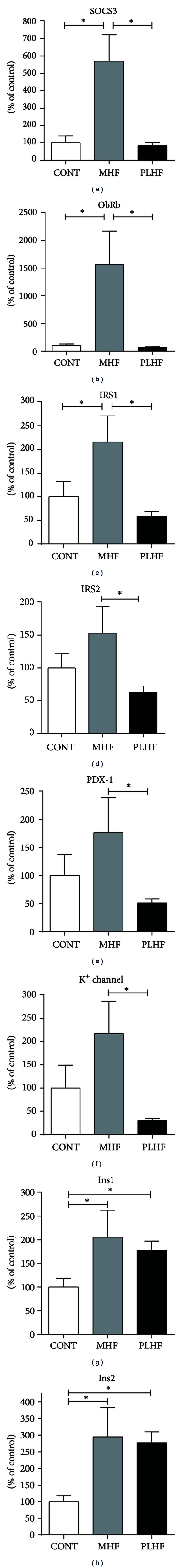
Pancreatic mRNA expression in adult CONT, MHF, and PLHF offspring. SOCS3: suppressor of cytokine signaling 3; IRS: insulin receptor substrate; PDX1: pancreatic and duodenal homeobox 1; ObRb: full length leptin receptor; Ins: insulin; Kir6.2: potassium channel subunit. Data are means ± SEM, *n* = 10–18 per group. *P < 0.05.

MHF offspring showed significantly higher levels of pancreatic mRNA levels of SOCS3, ObRb, and IRS1 compared to CONT and PLHF groups (Figure 3). mRNA levels of these genes were similar between CONT and PLHF offspring. Despite these similarities in expression levels between CONT and PLHF offspring, significant positive correlations that existed between these and other measured genes were primarily found in CONT and MHF offspring only and were not present in PLHF offspring (Table 2).

Levels of mRNA of IRS2, PDX-1, and Kir6.2 were significantly reduced in PLHF offspring compared to MHF offspring but were not different between any other groups. Pancreatic INS1 and INS2 expression was significantly increased in MHF and PLHF offspring compared to CONT (Figure 3).

**Figure 4:**
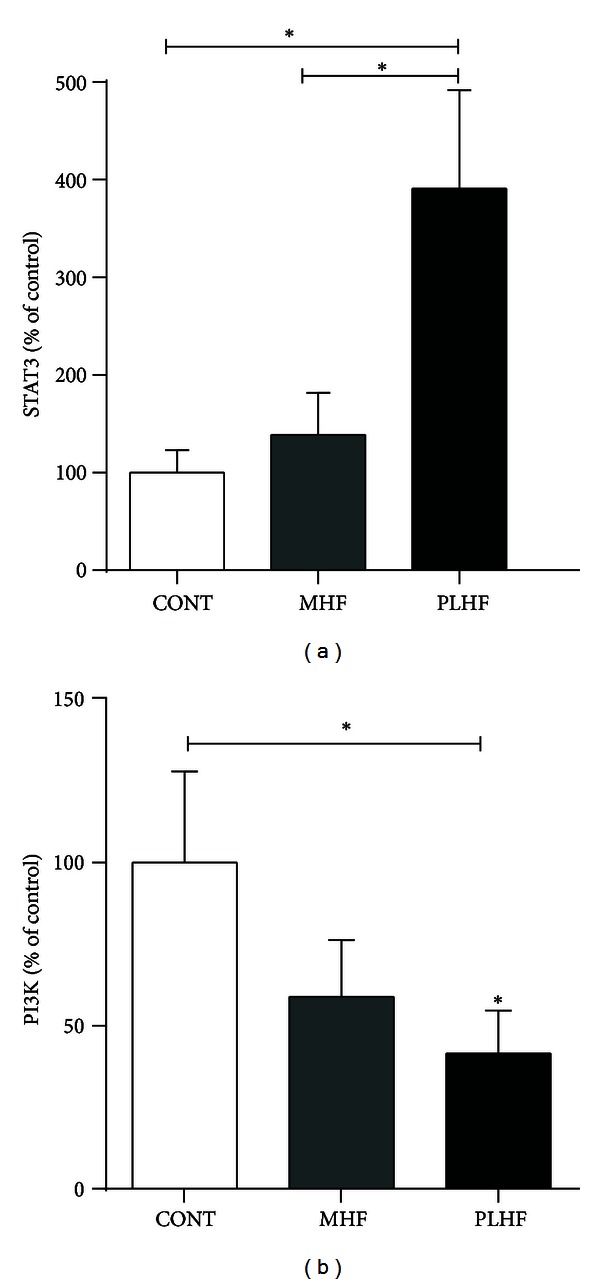
Pancreatic STAT3 (a) and PI3K mRNA expression (b). Data are means ± SEM, *n* = 10–18 per group. STAT3: signal transducer and activator of transcription 3; PI3K: phosphatidylinositol 3-kinase. *P < 0.05.

STAT3 expression was significantly increased in PLHF offspring compared to CONT and MHF offspring (Figure 4(a)). PI3K expression was significantly decreased in PLHF compared to CONT and MHF (Figure 4(b)). Of note, PI3K was positively correlated with IRS1 and IRS2 expression in PLHF offspring (*R* ^2^ = 0.44, *P* < 0.05 and *R* ^2^ = 0.72, *P* < 0.0005, resp.) but not in CONT or MHF offspring.

**Figure 5:**
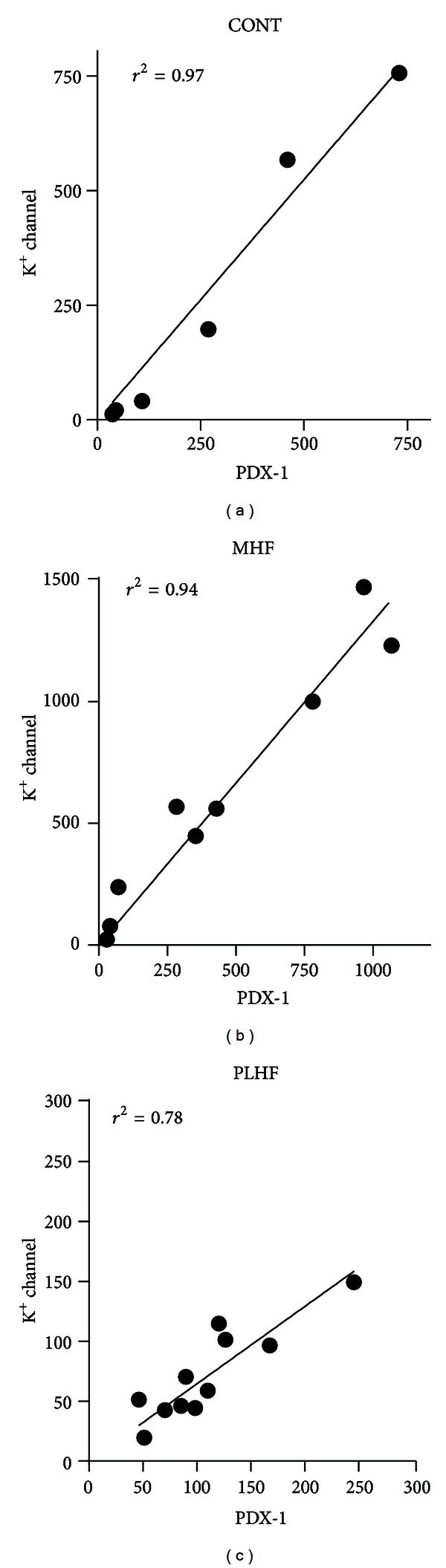
Relationship between PDX-1 and potassium channel subunit Kir6.2 in adult CONT, MHF, and PLHF male offspring. PDX-1, pancreatic, and duodenal homeobox 1. *N* = 8–10 per group.

**Figure 6:**
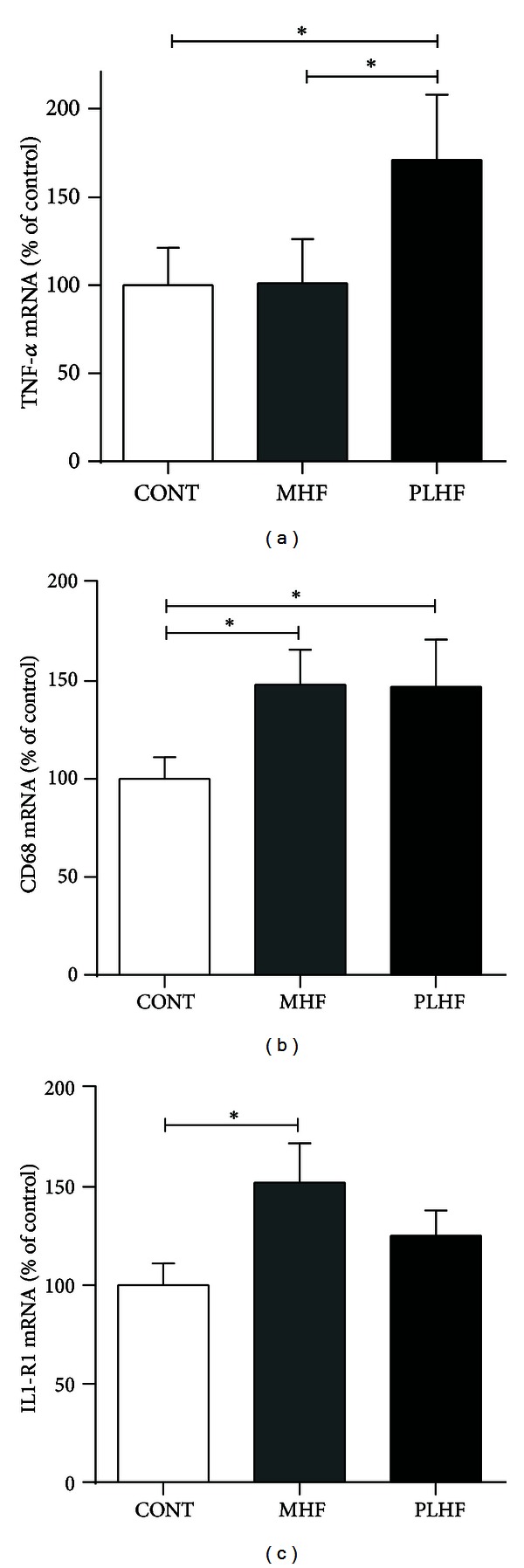
Pancreatic mRNA expression of TNF-*α* (a), CD68 (b), and IL1-R1 (c) in adult CONT, MHF, and PLHF offspring. TNF-*α*; tumor necrosis factor-alpha; CD68: cluster of differentiation 68; IL1-R1: interleukin 1 receptor, type 1. Data are means ± SEM, *n* = 10–18 per group. *P < 0.05.

Insulin receptor, leptin, ObRa, and phosphodiesterase 3B (PDE3B) were similar between groups (data not shown). A highly significant relationship was observed in all offspring groups between PDX-1 and ATP-sensitive potassium (Kir6.2) channel (Figure 5). Given the potential link between SOCS3 and inflammation, mRNA levels of key proinflammatory cytokines were also evaluated. There was an increase in TNF-*α* in PLHF offspring compared to CONT and MHF offspring (Figure 6(a)). The macrophage marker CD68 was increased in MHF and PLHF offspring compared to CONT (Figure 6(b)). There was an increase in IL-1R1 mRNA levels in MHF offspring compared to CONT offspring (Figure 6(c)). There were no differences in IL-6R, cardiotropin, IL-1B, GLUT2, GP130, CTNF, LIF, or TNF-R1 mRNA expression between any of the offspring groups (data not shown).

## 4. Discussion

There is clear clinical and experimental evidence that maternal obesity predisposes to obesity and metabolic disorders in offspring [14, 30]. However, despite the common phenotype of offspring obesity and hyperinsulinemia, the present study shows that at least in an animal model, that maternal nutritional history determines the pattern of mRNA expression of key regulatory genes in the pancreas of adult offspring. These data suggest that different regulatory pathways may lead to a similar metabolic phenotype and are in agreement with previous experimental studies suggesting that there may be separate influences of maternal obesity during the periconceptional period and late gestation on adiposity in offspring [31]. We have previously shown that “programmed” obesity and simple diet-induced obesity can develop via different underlying mechanisms [32]. The present study further reinforces the importance of the timing of the dietary insult on the metabolic derangements that ensue in offspring during adulthood.

SOCS3 inhibition of ObRb signaling has been proposed as a mechanism for leptin resistance in a range of tissues [33–35] including pancreatic *β* cells [36]. It is clear that leptin resistance in PLHF pancreata was mediated by mechanisms that did not involve SOCS3 and this has been reported previously in rat models of HF feeding [37, 38]. SOCS3 is normally increased at sites of both acute and chronic inflammation [39] and has been shown to specifically suppress STAT3 activation [40]. This agrees with the present study; a marked increase in SOCS3 mRNA levels in MHF offspring is paralleled by STAT3 levels similar to control offspring. Leptin is known to increase SOCS3 expression in isolated human pancreatic islets and in islets from ob/ob mice treated *in vivo* [36]; our data imply that leptin-induced SOCS3 stimulation appears intact in MHF offspring. Conversely, in PLHF offspring, SOCS3 expression levels were similar to controls in the presence of markedly elevated STAT3 expression. Of note, despite similar expression levels of SOCS3 between CONT and PLHF offspring, the positive correlations that were observed between SOCS3 and other key signaling genes were restricted mainly to CONT and MHF offspring only. This may point to a maladaptation in PLHF offspring due to the acute HF dietary exposure and a change in homeostatic set-points in MHF offspring due to mothers entering pregnancy in an already obese state. Despite the link between SOCS3 and inflammation, there were no observed changes in TNF-*α* mRNA levels in the MHF group whilst there was a significant increase in TNF-*α* expression in PLHF offspring. In addition, mRNA levels of the macrophage marker CD68 were increased in both HF groups and taken together may indicate differing macrophage phenotypes/polarization states resulting in divergent offspring inflammatory profiles dependent upon prior maternal nutritional background. Of note, despite marked changes in inflammatory markers at the tissue level, we observed no changes in circulating plasma TNF-*α* concentration. However, despite enhanced plasma IL-1*β* concentrations pancreatic IL-1*β* expression was unaltered between groups implicating other tissues as possible systemic sources of this proinflammatory cytokine.

PLHF offspring exhibited decreased PI3K mRNA levels compared to CONT animals. Induction of the ObRb/PI3K signaling pathway prolongs potassium channel opening, leading to decreased insulin exocytosis, a direct action of leptin which inhibits insulin secretion [41]. Moreover, the lowered PI3K mRNA levels in PLHF offspring were concomitant with a reduction in IRS1 and IRS2 mRNA, whose phosphorylation is coupled to PI3K activation. Of note, downstream of PI3K, PDE3B gene expression levels remained similar to controls, suggesting that altered leptin signaling was achieved in earlier stages of this pathway. Thus, attenuation of the ObRb/PI3K signaling pathway may mediate leptin resistance in these hyperleptinemic PLHF offspring. Such a mechanism has been observed before, in hypothalamic neurons of rats with diet-induced obesity (DIO) [42].

IRS proteins and PI3K have leptin independent roles in the *β* cell. Both are found downstream of the insulin receptor, in the IR/IRS/PI3K pathway which mediates the autocrine actions of the insulin-IR complex [43, 44]. Our observed attenuation of PI3K and IRS mRNA did not, however, affect insulin synthesis—PLHF animals were hyperinsulinemic, and preproinsulin mRNA concentrations were elevated. Furthermore mRNA concentrations of IR were unaffected potentially due to lack of SOCS3 induction in PLHF offspring, similar to Controls. Thus these mRNA changes of PI3K coupled to IRS likely reflect a mechanism of leptin resistance in this group.

Mean concentrations of ObRb mRNA in PLHF offspring were similar to controls, but a significant inverse relationship between plasma leptin and ObRb mRNA levels in the PLHF group was suggestive of ObRb downregulation. A downregulation of receptor populations in the face of elevated ligand concentrations is not uncommon in resistant states. It is seen, for example, in the hypothalamus in the presence of hyperleptinemia [45–47] and during pregnancy, which is a natural state of leptin resistance [48].

A rise in IRS1 and IRS2 mRNA associated with elevated ObRb and SOCS3 mRNA in MHF offspring may seem unexpected since SOCS3 is known to target insulin receptor signaling by interference with IRS action [49]. However, several studies have demonstrated that SOCS3 mediates insulin resistance in both adipocytes and hepatocytes through post-translational modifications such as inhibition of IRS tyrosine phosphorylation [50] and targeted ubiquitin-mediated degradation of IRS1 and IRS2 without effecting transcription [49, 51]. IRS proteins are involved in both leptin signaling and insulin signaling, so altered gene expression levels may represent changes in either (or both) pathways, although IRS response to leptin is reportedly much lower than that seen with insulin [52]. We observed an increase in IRS1 expression in MHF offspring compared to Controls. This upregulation of IRS1 expression may be related to the increased insulin production of the MHF offspring given the known role of IRS1 in the regulation of insulin secretion [53]. 

SOCS3 is also reported to repress preproinsulin gene transcription [41], although other mechanisms clearly countered this action in MHF offspring, since Ins1 and Ins2 mRNA expression levels remained elevated. This observation was confirmed by the significantly raised levels of circulating plasma insulin and the lack of any correlation in levels of mRNA between SOCS3 and Ins1 or Ins2.

Some of the observed differences may relate to the initial hyperphagia observed in the PLHF maternal group as reported previously [14]. Since rodents primarily eat on a calorific basis, they will passively over-consume calories when initially placed on an HF diet and self-regulate back to a normal total caloric intake over a period of a few days. It has been shown in other rodent models that alterations in nutrition around the time of implantation can lead to lasting metabolic effects in the offspring [54]. Maternal obesity has been shown to exert adverse effects as early as the oocyte and preimplantation embryo stage and that these effects may contribute to the lasting morbidity observed in offspring [55]. Similarly, even a very short period of altered nutrition (restricted to one ovulatory cycle) prior to natural mating can elicit lasting effects on the offspring [56] and this represents a further point of difference between the MHF and PLHF groups. 

The present study was not designed to incorporate the effect of a reversal paradigm whereby mothers fed the pre-conceptional HF diet were placed on a standard diet throughout pregnancy and lactation. However, while there is evidence that a period of dietary restriction in obese mothers may ablate the programming of obesity in offspring [57], it may be associated with an activation of the stress axis in the offspring [31]. Further, the data are inconsistent; it has also been shown that dams with diet-induced obesity, fed a standard diet throughout pregnancy and lactation, still transmit the propensity for obesity and metabolic disease to their offspring [58].

We recognize that gene expression analysis on whole pancreatic tissue is a limitation of the present study. Due to the size of the cohort and logistical considerations, only whole pancreatic tissue was a possibility, whereas ideally only pure islet populations would have been examined [59]. However, for the key genes presented such as ObRb, SOCS3, and IRS, expression has been shown to be primarily confined to the endocrine islets and not been detected in the exocrine pancreas [39, 41, 60]; therefore we are confident that the observed changes in the genes studied represent changes in the islets. Further, although we have shown that differences in pancreatic gene expression in offspring are dependent upon the timing of maternal high fat diet exposure, this does not itself imply causation in the development of the obese phenotype—other relevant pathways including liver, skeletal muscle, and fat metabolism will also be integrated into the programmed phenotype development.

This study has demonstrated enduring deleterious effects of a maternal HF diet during early windows of development. Maternal HF feeding both before conception and/or through pregnancy and lactation induced obesity, hyperinsulinemia, and hyperleptinemia in adult offspring, even though offspring were fed a standard diet postnatally. Timing of exposure to the maternal HF diet differentially altered gene expression in signaling pathways downstream of the pancreatic leptin receptor that mediated leptin resistance. This also raises the possibility that despite common offspring obesity, treatment modalities may only be efficacious in a subpopulation of treated offspring as the current data suggest that the metabolic pathways disrupted will be dependent upon the timing of the early life dietary insult.

## Figures and Tables

**Table 1:** Sequences of forward and reverse primers.

	Entrez gene ID	Forward primer	Reverse primer
K^+^ channel (Kir6.2)	83535	5′-GAA GGA GGC AAA TGA TTG GA-3′	5′-AGT GTC CCC CAG ACA AAG TG-3′
PDE3B	29516	5′-GAC CGT CGT TGC CTT GTA TT-3′	5′-CGA TCG CCT TTC TCT ACT GG-3′
SOCS3	89829	5′-TTC TTT ACC ACC GAC GGA AC-3′	5′-CGT TGA CAG TCT TCC GAC AA-3′
Insulin1	24505	Commercially prepared primer, QuantiTect Primer Assay, Cat. no. QT00373303 (Qiagen)	
Insulin2	24506	Commercially prepared primer, QuantiTect Primer Assay, Cat. no. QT00177380 (Qiagen)	
Insulin receptor	24954	5′-ATC CGT CGC TCC TAT GCT CT-3′	5′-TCG TGA GGT TGT GCT TGT TC-3′
Leptin	25608	Commercially prepared primer, QuantiTect Primer Assay, Cat. no. QT00190960 (Qiagen)	
ObRa	24536	5′-TGA TAT CGC CAA ACA GCA AA-3′	5′-AGT GTC CGC TCT CTT TTG GA-3′
ObRb	24536	5′-AAA GCC TGA AAC ATT TGA GCA TC-3′	5′-CCA GAA GAA GAG GAC CAA ATA TCA C-3′
Pdx1	29535	Commercially prepared primer, QuantiTect Primer Assay, Cat. no. QT00405328 (Qiagen)	
STAT3	25125	Commercially prepared primer, QuantiTect Primer Assay, Cat. no. QT00183512 (Qiagen)	
STAT5B	25126	Commercially prepared primer, QuantiTect Primer Assay, Cat. no. QT00192024 (Qiagen)	
CD68	287435	Commercially prepared primer, TaqMan expression assay, Cat. no. Rn01495634 (Applied Biosystems)	
TNF-*α*	24835	Commercially prepared primer, TaqMan expression assay, Cat. no. Rn01525859 (Applied Biosystems)	
Cyclophilin	25518	5′-TTG GGT CGC GTC TGC TTC GA-3′	5′-GCC AGG ACC TGT ATG CTT CA-3′
HPRT	24465	5′-AGT CCC AGC GTC GTG ATT AG-3′	5′-CCC CCT TCA GCA CAC AGA-3′

**Table 2:** Correlations between SOCS3 and other key genes. *N* = 8–13 per group.

	CONT	MHF	PLHF
SOCS3 versus ObRb	*R* ^2^ = 0.69, *P *= 0.08	**R** ^2^ ** = 0.87, P = 0.0008**	*R* ^2^ = 0.03, *P* = 0.64
SOCS3 versus IR	**R** ^2^ ** = 0.67, P = 0.02**	*R* ^2^ = 0.02, *P* = 0.71	*R* ^2^ = 0.002, *P* = 0.90
SOCS3 versus IRS1	**R** ^2^ ** = 0.84, P = 0.01**	**R** ^2^ ** = 0.58, P = 0.02**	*R* ^2^ = 0.04, *P* = 0.51
SOCS3 versus IRS2	**R** ^2^ ** = 0.98, P = 0.002**	**R** ^2^ ** = 0.58, P = 0.02**	*R* ^2^ = 0.15, *P* = 0.21
SOCS3 versus Kir6.2 channel	**R** ^2^ ** = 0.82, P = 0.03**	**R** ^2^ ** = 0.93, P < 0.0001**	*R* ^2^ = 0.002, *P* = 0.90
SOCS3 versus Pdx1	**R** ^2^ ** = 0.78, P = 0.009**	**R** ^2^ ** = 0.91, P = 0.0003**	*R* ^2^ = 0.07, *P* = 0.40

## Abbreviations

CD68: Cluster of differentiation 68
CONT: Control
DEXA: Dual energy X-ray absorptiometry
ELISA: Enzyme-linked immunosorbent assay
GLUT2: Glucose transporter 2
GP130: Glycoprotein 130
HF: High fat
HOMA: Homeostatic model assessment
HPRT: Hypoxanthinephophoribosyltransferase
IL: Interleukin
Ins: Insulin
IR: Insulin resistance
IRS: Insulin receptor substrate
Kir6.2: Potassium channel subunit
LIF: Leukemia inhibitory factor
MHF: Maternal high fat
OBR: Leptin receptor
PDE3B: Phosphodiesterase 3B
PDX-1: Pancreatic and duodenal homeobox-1
PI3K: Phosphatidylinositide 3-kinase
PLHF: Pregnancy and lactation high fat
SOCS3: Suppressor of cytokine signaling 3
STAT3: Signal transducer and activator of transcription 3
TNF-*α*: Tumor necrosis factor-alpha.
